# Development and Validation of a Persian Version of Dichotic Emotional Word Test

**Published:** 2016-03

**Authors:** Atefe Davudzade, Abdolreza Shaibanizadeh, Zahra Jafari, Farzin Fahimnia, Masoud Haghani

**Affiliations:** 1 *Department of Audiology, School of Rehabilitation Sciences, Iran University of Medical Sciences, Tehran, Iran.*; 2 *Rehabilitation Research Center, School of Rehabilitation Sciences, Iran University of Medical Sciences, Tehran, Iran.*; 3 *Department of Linguistics, Linguistics Faculty, Institute for Humanities and Cultural Studies, Tehran, Iran.*; 4 *Department of Biostatistics, School of Management and Medical Information Science, Iran University of Medical Sciences, Tehran, Iran.*

**Keywords:** Dichotic Listening Test, Emotional Aspects, Right Hemisphere, Cerebral Dominance

## Abstract

**Introduction:**

Emotional words in comparison with neutral words have different hemispheric specialization. It is assumed that the right hemisphere has a role in processing every kind of emotional word. The objective of the present study was the development of a Persian version of the dichotic emotional word test and evaluate its validation among adult Persian speakers.

**Materials and Methods:**

The present study was done on 60 adults, with the age ranging from 18-30 years for both genders, who had no history of neurological disorders with normal hearing. The developed test included eight main lists; each had several dichotic emotional/ neutral pairs of words. Participants were asked to recall as many words in each list as they could after they listened to them. A content validity index was used to analyze the validity of the test.

**Results:**

The mean content validity index score was 0.94. The findings showed that in the left ear, emotional words were remembered more than neutral ones (P=0.007). While in the right ear, neutral words were remembered more (P=0.009). There were no significant differences in male and female scores.

**Conclusion:**

Dichotic emotional word test has a high content validity. The ability to remember emotional words better in the left ear supports the dominant role of the right hemisphere in emotional word perception.

## Introduction

Feelings and Emotions are an inseparable part of life, which affect behavior, making decisions, motivation, and social interactions (1). Emotional words are special words which are processed in a faster and more complicated way compared to neutral stimuli. Emotional words (like hate and love), in comparison with neutral words, result in more contrasted responses (2). Cognitive functions like language and logic have apparently been accepted as being within the scope of the left hemisphere, while the right hemisphere’s involvement in emotional functions is not that certain (3). The findings of different studies have proposed three main hypotheses for hemispheric lateralization in emotional word processing. One of these findings corroborates the specialization of the right hemisphere model for emotional processing (whether positive or negative) which is possibly due to the increased contribution of the right hemisphere in mechanisms of autonomic and behavioral arousal (Right Hemisphere Hypothesis) (4-6). Some studies also suggest a different hemispheric asymmetry. In this model, positive emotion is more processed in the left hemisphere while negative emotion is more processed in the right one (Valence Hypothesis) (7,8). Other research indicated that the left hemisphere has a key role in processing emotional information (Left Hemisphere Hypothesis) (5,9). 

In spite of the various hypotheses in processing and perception of emotional words, most studies support the right hemisphere hypothesis (3). For instance, Bower and Heilman (1990) studied sentences which were different in their propositional content. These sentences were presented in four different emotional intonations (happy, sad, angry, and indifferent) to two groups of patients, one with a right hemisphere infarction and the other with an infarction in the left hemisphere. Patients with damage in the right hemisphere, compared with the other group, had worse results in determining the speakers’ emotional tone (10). In another research, Ortigue et al (2004), by using electroencephalogram, worked on stimuli processing of positive, negative, and neutral words. The findings of this study showed that the words presented in the left visual field (right hemisphere) would be detected better, if they had emotional load (11). In another study, in order to investigate the ability of evaluating emotional facial expressions,Geoffrey et al(1991),by injecting sodium amital, found that by inactivating the right hemisphere (nonlinguistic hemisphere), the ability of patients in distinguishing emotional gestures was less than moderate (12). But Pell et al (2006), in their research, found that the processing of emotional tone takes place in the two hemispheres. Eviatar et al (1990) also obtained no evidence to prove valence and right hemisphere models (4). However, both the two recent studies were conducted with brief stimuli without considering a memory component (4). 

It is assumed that in emotion processing many cortical and sub-cortical components are involved, which generally have some roles in activities like cognition, memory, and emotion (13,14). Because of the lack of accessibility to these parts for functional assessment, most of the studies have dealt with the general role of the cerebral hemispheres in the processing of emotional information. In recent years, researchers’ attention toward the assessment of emotional stimuli perception in different normal and pathological groups has been increased. To achieve this, they have utilized different methods including tools related to the evaluation of the central auditory system. One of the common ways to evaluate the performance of cerebral hemispheres is using dichotic listening tests. These tests which are a type of working memory test are one of the most valid behavioral central auditory processing tests for evaluation of the cerebral hemispheres’ functions, transferring information between the two hemispheres, growth and maturation of the neural auditory system, and diagnosing central auditory deficits. These tests are based on simultaneous presentation of various auditory stimuli to the two ears (15,16). Since the right hemisphere has more responsibility in processing the presented stimuli to the left ear, it is assumed that presenting the emotional stimuli to the left ear has better responses and thus neutral or non-emotional words are better remembered when presented to the right ear. Therefore, by using dichotic listening tests, the role of right hemisphere in emotional word processing is more specifically determined. Most of the studies in the field of the lateralization of emotion were conducted by visual tools (4,11,12) and the studies that utilized a dichotic listening tool focused on the musical aspect of speech such as pitch, length, and loudness of vocalization to benefit from prosodic features and not on lexical content (3). 

In 2005, the dichotic emotional word test was developed by Chong Sim and Martinez and was conducted on 62 English right handed participants (3). The Persian version of the present test has been completely developed based on the above-mentioned study. Regarding the lack of similar tests in Persian, in the present study this test was developed by using sets of emotional and neutral words with a particular focus on lexical content of words among adult Persian speakers. Then the content validity of the test was evaluated.

## Materials and Methods

This study includes three main stages: the development of the Persian version of the dichotic emotional test, evaluation of content validity and finally its administration on participants who met inclusion criteria. The present set of stimuli has been made up of neutral and emotional word lists. At first, a list of 180 words was selected among the most frequent words by using three Persian frequency dictionaries. In this list, the number of neutral and emotional words was the same. Also negative and positive emotional words in the list were equal in number. Being simple and meaningful was considered for the selection of the words. In addition, being abstract was also taken into account for the selection of the neutral words. Finally, 148 selected core words were grouped in 10 lists which included 8 main lists and two practice lists. Each main list consisted of 8 pairs of dichotic words. Each list had the same number of neutral and emotional words and each pair of dichotic words included one neutral and one emotional word. The linguistic criteria taken into consideration to make pairs of dichotic words are having the same number of syllables, length of syllables, frequency of words, and being formal or informal. Furthermore, each word shouldn’t have any phonetic and/or semantic balance with the rest of the words in each list. To diminish the effect of strong semantic relation between negative or positive emotional words that may result in improving the remembered words, they were placed in an every-other-word way. Practice lists were composed of 5 pairs of words.

After finalizing the lists, the developed test was given to 10 experts (including 4 audiologists, 2 linguists and 4 speech therapists) in order to validate its content. The experts scored each list based on a four-point scale where 1 equals not meeting the selected criteria, 2 some modifications are needed, 3 some minor modifications, and 4 meeting all the selected criteria. To ensure the content validity of a study, different methods can be used. Recently, one of the common methods is computing the CVI (17-19). By comparing different methods of ensuring the content validity, Polit et al (2007) demonstrated that calculating the CVI has more advantages compared with other present methods. Being simple and understandable are the most important advantages of this method (20).

Each word was articulated by a male speaker with a neutral tone (without prosody), and then recorded on a CD. The recorded words were saved in Audacity Software (2.0.6 version). At the first stage, each word was put in pairs with another word in a dichotic way so that the neutral word be presented on one channel and the emotional word on the other. The onset of the two words in each pair was simultaneous and one second pause was placed between each pairs of words. At the beginning and end of each list, a 1000Hz pure tone with a duration of 100ms with a constant intensity level was placed. In fact, this tone is a sign to start each list with. One second after the first pair of dichotic words and one second after finishing the final pair of dichotic words, the same binaural signal was presented. 

There were two alternatives in presenting pairs of words: emotional words to the left ear (EL) and neutral words to the right ear (NR), and emotional words to the right ear (ER) and the neutral words to the left ear (NL). 

In the final step, the test was administered on 60 participants (30 male and 30 female), in the age range of 18 to 30-years-old. All participants had a normal hearing threshold (15 dB HL or better within the frequency range of 500-4000Hz). Edinburg handedness questionnaire was administered and only those with 100% right-handedness were selected to be in the study. Being monolingual in Persian and not having a history of neurological or psychiatric diseases and drug or alcohol abuse were the inclusion criteria for the participants. The present study was approved by the ethics committee of Iran University of Medical Sciences, Tehran, Iran. The test stimuli were presented by using a laptop in each participant’s MCL (Most Comfortable Level) in a dichotic manner. They were sitting behind a desk with a laptop on it (the background noise was minimum). First, they were given two practice sets. These sets continued until they were able to learn the instructions. In order to decline the serial effect in the responses, they were asked to do a distracter task (10 items of simple math problems) after hearing the final signal. Then, immediately after the distracter task, they had one minute to write the remembered words. The percentage of the correct emotional and neutral words remembered in each ear was considered as the participant’s score. The beginning of the test for half of the participants was with emotional words to the right ear and neutral words to the left ear and vice versa for the other half of the participants (i.e.,the emotional word to the left ear and the neutral word to the right ear).

Statistical analysis in the present study, the content validity ratio (CVR) was calculated by the following formula for measuring the test validation. Then, the content validity index (CVI) was computed. According to the previous studies, the comments of 3-10 experts with a CVI higher than 0.75 are considered as an accepted level (17-19). 

CVR= (ne-N/2) /N/2

‘Ne’ refers to the number of 3and4 option/alternative/item chosen by the experts and ‘N’ refers to the number of all experts (21). The normal distribution of data was done by Kolmogorov-Smirnov (P≥0.156).

 Hence it was not meaningful, parametric statistics were used. To compare the mean of the participants’ scores in the left and right ears, paired sample t-test and independent sample t-test were employed. And to study the gender effect, independent sample t-test was employed. The level of meaningfulness was considered 0.05 for all variables. All analyses were done by SPSS Software version 21. 

## Results

The content validity index for the whole test is 0.94. Table 1 shows the CVI for each of the eight main lists. 

**Table 1:** CVI* Scores for 8 word lists

**Lists**	1	2	3	4	5	6	7	8
**CVI**	0.97	0.97	1	0.97	0.87	0.97	0.97	0.97
*CVI: content validity index								

Gender and the direction of the beginning of the test (two conditions) had no effects on the results. As indicated in table 2, the percentage of the correct emotional words was more when they were presented to the left ear (with the mean of 39.78 percent for the left ear and 35.96 percent for the right one). While the neutral words were better remembered when they were presented to the right ear (with the mean of 39.46 percent for the right ear and 36.03 percent for the left one).

According to the results shown in table 2, the difference between the mean score of emotional word perception compared with the neutral word perception was statistically significant in the left ear (P=0.007) and right ear (P=0.009). However, the difference between the mean score of the perception of all the words in the right ear in comparison with the left ear was not statistically significant (P=0.85). There also was no significant difference between the scores of the lists (P≥0.156).

**Table2:** Means and standard deviations of percentage of correct responses by stimulus Type

Mean ± SD* (%)	**Stimulus Type**
Emotion Words in Left	39.78 (7.90)
Emotion Words in Right	35.96 (8.25)
Non-emotion Words in Left	36.03 (8.68)
Non-emotion Words in Right	39.46 (9.68)
All Words in Left	37.90 (6.53)

## Discussion

The results of the present study in the emotional and neutral word perception in the left and right ears have revealed that the emotional words were better remembered in the left ear rather than the neutral words (without any difference in recalling positive and negative words). Also, the neutral words were better remembered in the right ear. With regard to the right hemisphere hypothesis, which claims that the right hemisphere has a more dominant role in emotional word processing (whether positive or negative). In addition, considering the fact that auditory information processing of the left ear is done in the right hemisphere supports that the emotional word perception happens in the right hemisphere. The result of this research is in harmony with Sim and Martinez (2005) and the majority of studies in this field. For example, Almuhammadi and Alzahrani (2013) conducted a research with the aim of determining hemispheric lateralization in the emotional processing of auditory information in Arabic speakers. Their findings showed the significant left ear advantage in emotional processing. In their study, similarly to the present study, the relation between ear advantage and gender was not significant (22). Nagae and Moscovitch (2002) by using visual stimuli demonstrated that explicit memory of emotional words is mostly related to the right hemisphere (3). Kuchinke et al (2005) studied emotional word processing with FMRI in normal right-handed participants. The findings showed the dominant role of right inferior frontal gyrus in processing this kind of stimuli (23). 

Although in previous studies, emotional word perception and subsequently right hemisphere advantage have been investigated

in normal groups; they were different in stimuli presentation manner, test tasks, and the way of responding. For example, most of the tests in this field used visual stimuli to study emotional perception, while processing manner under the observation conditions affects results patterns (6). On the other hand, since the material of the present test is of a word type, it is expected that the left hemisphere advantage in word processing (verbal memory), masks to some extent the right hemisphere advantage in emotional word processing (4). So it can be the cause of the contradiction which exists in the findings of previously done studies with verbal materials. The type of requested task also can change the results remarkably. Activities requiring word identification, to some extent obscure right hemisphere contribution in emotional processing (because of the main role of left hemisphere in the early perceptual processing of words) and so make its identification difficult. Hemispheric differences in emotional processing, more probably appear in the later stages of processing (at the time of analysis of high-order attributes such as emotional content). By choosing the type of task which can involve memory, the right hemisphere contribution in emotional processing becomes clearer (4, 24). The research which investigated the hemispheric differences in terms of memory of emotional and neutral words were rare (5). In a normal dichotic listening task, a pair of stimuli is presented and participants should keep them in minds for a short period of time before responding. This means that dichotic listening, like other activities which engage the memory, also needs encoding, storage and retrieval (25).

In general, the findings of the present study and the majority of the previous studies underlined the right hemisphere hypothesis. However, it seems that factors like the emotion of a word, task type, way of processing (information perception, expression and experience) and type of stimuli presentation (visual/ auditory) influence the results’ pattern (4,6,24). The emotional content of the words in this test caused left ear advantage (although no emotional prosody existed in the words). This finding also provides strong support for the right hemisphere hypothesis in forming a stronger memory for the emotional words presented to the left ear (3). This test, both as a whole and in each list of words, has a high degree of content validity.

The perception of feelings and emotions is influenced in many diseases like brain damages, mental retardation, and psychiatric illnesses such as schizophrenia and depression. Regarding the advantages of the present test, that is a simple, non-invasive, low cost and available test (25), it can be used as a practical and clinical test. 

## Conclusion

This study attempted to develop the Persian version of the dichotic emotional word test. Meanwhile, it has provided the support for the importance of the right hemisphere (left ear) role in emotional word perception. Moreover, it provides a validated test aiming at helping in the diagnosis and improvement of the processing deficits in emotional fields to experts and therapists. Currently this study’s researchers work on its reliability evaluation and constituent validity. It is hoped that the outcome would effectively help to apply this test to a wide range of neurological disorders in the near future.
